# Local adaptation to continuous mowing makes the noxious weed *Solanum elaeagnifolium* a superweed candidate by improving fitness and defense traits

**DOI:** 10.1038/s41598-021-85789-z

**Published:** 2021-03-23

**Authors:** Jesus Chavana, Sukhman Singh, Alejandro Vazquez, Bradley Christoffersen, Alexis Racelis, Rupesh R. Kariyat

**Affiliations:** 1 Department of Biology, The University of Texas Rio Grande Valley, Edinburg, TX 78539 USA; 2 School of Earth, Environmental and Marine Sciences, Edinburg, TX 78539 USA

**Keywords:** Ecology, Invasive species

## Abstract

The role of disturbance in accelerating weed growth is well understood. While most studies have focused on soil mediated disturbance, mowing can also impact weed traits. Using silverleaf nightshade (*Solanum elaeagnifolium*), a noxious and invasive weed, through a series of field, laboratory, and greenhouse experiments, we asked whether continuous mowing influences growth and plant defense traits, expressed via different avenues, and whether they cascade into offspring. We found that mowed plants produced significantly less number of fruits, and less number of total seeds per plant, but had higher seed mass, and germinated more and faster. When three herbivores were allowed to feed, tobacco hornworm (*Manduca sexta*) caterpillars, gained more mass on seedlings from unmowed plants, while cow pea aphid (*Aphis craccivora*), a generalist, established better on mowed seedlings; however, leaf trichome density was higher on unmowed seedlings, suggesting possible negative cross talk in defense traits. Texas potato beetle (*Leptinotarsa texana*), a co-evolved specialist on *S. elaeagnifolium,* did not show any differential feeding effects. We also found that specific root length, an indicator of nutrient acquisition, was significantly higher in first generation seedlings from mowed plants. Taken together, we show that mowing is a selective pressure that enhances some fitness and defense traits and can contribute to producing superweeds.

## Introduction

Weeds are generally defined as undesired plant species that can invade ecosystems, causing harm to both biotic and abiotic ecosystem components^1,2^. The factors contributing to the ability of weedy plant species to establish and colonize have been well understood^3–5^. The general consensus is that weed species tend to have enhanced traits that allow them to succeed either in their native or introduced habitats, when compared to their non-weedy counterparts^6–8^. This could be their ability to either outcompete heterospecifics and/or have better growth, fitness, and defense traits to name a few^9^. For example, allelopathy in weedy sunflower (*Helianthus spp.*) inhibits mustard (*Brassica spp*.) seed germination and lantana (*Lantana camara*) inhibits wheat (*Triticum aestivum*), soybean (*Glycine max*), and corn (*Zea mays)* growth^10,11^. On the other hand, weeds such as *Rhododendron ponticum* and *Rhododendron maximum* colonize forests by adaptative switching between sexual and asexual reproduction, thereby reducing tree growth and regeneration, causing immense forest damage. They are also more tolerant to cold and shade and express plasticity in morphological and physiological adaptions to varying environmental conditions^12^. More recently, there has been tremendous interest to identify and quantify other contributing factors to weed success including their ability to cope with climate change, and more importantly, human disturbance^13–15^. However, most of these studies are limited to traits observed in a single growing season, ignoring any possible cascading effects.

The effects of human disturbance on weed success have also been well researched. Collectively, these studies suggest that land and soil disturbance due to human activities tend to enhance weed success both in natural and agricultural environments^16–18^. Many plants that thrive in hot and dry environments tend to become weedy with fast growth and drought resistance, primarily facilitated and enhanced by human disturbance^19^. These can range from clearing, draining, and other human activities that promote erosion, collectively damaging non-weedy vegetation^4^. Recently, in the semi-arid open forest with *Prosopis caldenia* (Caldenal), anthropogenic disturbance (e.g., fire, grazing) played a significant role in the establishment of widely distributed ruderal weed specie^20^. In rhizomatous weeds such as Carolina horsenettle (*Solanum carolinense*; Solanaceae), a single mother plant can produce ~ 21 new sprouts in the following season, a grave concern to farmers when rhizomes are broken apart in agricultural lands as part of tillage^21^. While obvious human-driven disturbances have been studied extensively among management practices, mowing as a disturbance has been overlooked, even though mowing is known to dramatically reduce photosynthetic area and reduce biomass, forcing them to reallocate resources^22–24^.

Weeds in urban, agricultural, and other forms of managed systems undergo multiple mowing events during their growing season and must constantly reprogram growth, defense and fitness, else risk extinction in local populations^25,26^. For example, Yong et al. demonstrated in the invasive weed *Erigeron annuus*, mowing reduced seed mass but led to variation in pappus length, and achene size, and speculated that these differences lead to better spread and higher survival rate^27^. Moreover, mowing is also considered as mechanical wounding, leading to enhanced defenses, both locally and systemically with short and long-term effects^28,29^. This interplay of anthropogenic disturbance and weed ecology, and its role in cascading growth and defense traits needs to be better understood, especially since many weed species are perennial and can propagate asexually over multiple years and growing seasons^21^.

Clearly, anthropogenic disturbances have a huge impact on weed success, and we are yet to understand the factors that contribute to this. Plants are known to evolve in short periods of time in response to environmental changes including temperature and CO_2_ levels, and selection favors genotypes with traits capable of surviving such stressors. These include growth, defense, resource allocation, flowering and reproduction, and germination rate is considered as one of the most important factors that indicate successful adaptation against environmental vagaries^30,31^. Among the many weedy traits that provide an edge over non weedy plants, the ability to self-fertilize is also considered critical^32,33^. In line with Baker’s theory^34^, it is observed that self-fertility is common in weeds, and the ability to self-pollinate and set viable seeds ensures fitness in founding populations, when cross pollen from conspecifics may be low, due to small population size and reduced number of unrelated individuals^35,36^. While most studies have addressed these questions using fitness measures as variables of interest^21,37^, we still lack a complete understanding of whether other biotic and abiotic factors can contribute and complicate the interactions at multiple trophic levels. In their home ranges, weeds are constantly subject to high herbivory pressure from co-evolved herbivores, a phenomenon lacking in their invasive habitats- commonly known as enemy free space. For example, the tropical fire ant, *Solenopsis geminata (*Frabicius), and the Asian house rat, *Rattus tanezumi* Temminck, are widely known to consume weed seeds of *Digitaria ciliaris*, *Echinochloa colona* and *Eleusine indica* and control weed populations in rice fields^38^ in their native ranges.

A large body of work has demonstrated that in the absence of these natural predators, weeds in an enemy free space are more likely to thrive and become a larger problem as they are highly invasive^39–42^. In addition, there is the possibility for local herbivores that differ in specialization and feeding guild to also impact these defense traits and weed fitness, by checking weed populations at an ecosystem threshold. For example, we previously found that in *S. carolinense,* intraspecific variation due to experimental inbreeding affected the recruitment of herbivores and natural enemies in field, by selectively improving fitness of outbred progeny when compared to inbred through better defenses, in addition to better growth and reproductive fitness^43^ with transgenerational effects^44^. Expanding this line of research into anthropogenic disturbance is critical to determine the evolutionary ecology, an area traditionally under-explored, but has gained momentum recently^43,44^. Being the chief contributor of fitness, seeds are loaded by mother plants with nutrients which have direct effects on offspring success^45^. Parental stress can lead to depleted resource allocation to seeds as a result of the lack of resources due to photosynthetic tissue loss by herbivory, or reallocation and tradeoffs at defense-fitness traits^46–48^.

To examine reproductive fitness, defenses, and possible local adaptation due to mowing, we used a combination of field, growth chamber, common garden, and lab experiments with multiple genets from 4 mowed and unmowed sub populations of Silverleaf nightshade (SLN) to ask the following questions: (1) Does mowing influence growth and fitness traits, (2) On what scale does mowing affect herbivore incidence and field damage, (3) Does mowing lead to local adaptation on growth and fitness traits, (4) Are there cascading effects on plant defenses against generalist and specialist herbivores, and (5) Are these effects if any, also prevalent in root traits, since the species also reproduce through rhizomatous roots?

We hypothesized that due to consistent loss of photosynthetic area and growing time, mowed plants will exhibit lower growth and fitness traits, but have enhanced defenses since mowing is regularly inflicted and is also a form of mechanical wounding and can lead to defense signaling and local adaptation. We also hypothesized that offspring, from mowed mother plants will have compromised growth and fitness traits due to lower resource allocation but will also have higher constitutive defenses due to damage in the parental generation. To answer these questions, we used three herbivores-. Tobacco hornworm (*Manduca sexta*), a generalist on Solanaceae, cow pea aphid (*Aphis craccivora*) a generalist aphid and Texas Potato Beetle (*Leptinotarsa texana*), a co-evolved specialist on SLN for our herbivory experiments. And, to examine root traits, we used WinRhizo Pro 2019 root scanner to measure the key root traits involved in weed success.

## Materials and methods

### Study system

SNL is a noxious, drought-resistant, perennial weed that is believed to have originated in the southwestern border of the United States and Mexico^49^ but is invasive worldwide^50^. The species thrives well in all environmental conditions including poor soil and nutrient availability^51^. It can easily spread to other locations by rivers and streams, through livestock manure and anthropogenic activity such as plowing and mowing^52^. The species also exhibits allelopathy, physical and chemical defenses, and high reproductive fitness by seeds and asexual reproduction through rhizomes, collectively making it highly competitive and extremely invasive^53,54^. Additionally, the species exhibits gametophytic self-incompatibility, but is also plastic for the trait, producing selfed seeds when outcrossed pollen is limited^55^.

### Study populations and plant materials

For all the experiments detailed in this study, we used plants and seeds derived from 8 locations in the McAllen-Edinburg area of Rio Grande Valley, Texas, USA where SLN is native. We had been monitoring these locations in the McAllen-Edinburg city limits for over three years^56^ and have confirmed the disturbance status of these populations; mowing has been done by city management continuously and 4 out of eight populations were disturbed by continuous mowing by the city management, and the rest were left undisturbed, but within 20–30 m from each other. The GPS coordinates and population size of these locations have been detailed in Supplementary Table 1.

**Table 1:** Details of response variables, treatment means, statistical tests and significance from WinRhizo experiment.

Response variable	Mean and SE (mowed)	Mean and SE (un mowed)	T value	P value
Shoot length	21.8 ± 1.6	19.3 ± 1.2	1.24	0.225
Root width	23.46 ± 0.93	25.0 ± 0.73	− 0.130	0.204
Root height	16.12 ± 0.63	17.23 ± 0.35	− 1.54	0.138
Root length	1142 ± 110	1623 ± 104	− 0.91	**0.004**
Total surface area	182.9 ± 22	257.7 ± 20	− 2.54	**0.017**
Root volume	2.45 ± 0.39	3.38 ± 0.40	− 1.66	0.108
Root tips	7928 ± 1150	15,447 ± 1904	− 3.38	**0.003**
Root forks	13,483 ± 1700	19,381 ± 1569	− 2.55	**0.017**
Root crossings	1148 ± 131	1563 ± 144	− 2.14	**0.042**
Pooled fine roots	9268 ± 1256	17,349 ± 2000	− 3.42	**0.0002**

Variables that are statistically significant are in bold P values at P < 0.05.

### Fruit collection

All the fruits produced by ~ 100 different plants (genets) with at least ten genets from each location were collected over one week in December 2019. The mowing (4 rounds of mowing) ended in late summer (September) and so the plants could set and mature fruits. Care was taken to ensure that the genets were at least 5 m apart to minimize any clonality effects in sampling since SNL can vegetatively reproduce through rhizomes. The collected fruits, from different locations in the city, were pooled per genet, bagged and stored at room temperature in lab for seed extraction.

#### Seeds/fruit and total seeds

Seeds were extracted from the fruit by cutting each fruit in half and gently squeezing the fruit to push out all the seeds into a fine mesh strainer (250 microns). Water was used to remove the pulp of the fruit from the seed. Once washed, seeds were set out to dry on a paper towel at 70 °C and 50% relative humidity for 24 h.

#### Fruit diameter and seed mass measurements

To measure fruit diameter and seed mass, five random fruits from the pooled fruits of a single genet (individual plant in each sub population/treatment i.e., from each location of mowed and unmowed areas) were chosen. Fruit diameter was measured using a digital caliper (ABSOLUTE Super Caliper SERIES 500, IL, USA). Afterwards, the fruits were carefully cut in halves without causing any damage to seed. The fruit’s pulp was removed, and the seeds were separated using a fine mesh strainer and then were dried on a napkin for at least 12 h. Once dried, the seeds were counted and stored in falcon tubes at 70 °C and 50% relative humidity.

To create a seed bank from each location (each field area of mowed and unmowed plants) for germination experiment and mass measurement, 200 seeds were randomly chosen from the pooled seeds of all the fruits from each genet per location. 100 of these seeds from both treatments (mowed and unmowed) from each of the eight locations were then weighed using an analytical balance (Accuris Dx W3101A-220, Mid Sci, MO, USA) to get 100 seed mass.

#### Seed germination and establishment

To examine seed germination and seedling establishment, 200 seeds from each of the pooled seed banks (from all eight locations) were used (1600 seeds in total; 800 per treatment). Before sowing, half of the seeds (400 each from each treatment) were treated with 20 ml of gibberellic acid in deionized water (mowed/unmowed) (GA_3_, 1000 ppm; Sigma-Aldrich, MO, USA) for 24 h to examine whether treatment × seed germination is impacted by the rooting hormone^57^. After the seed treatment, 50 seeds each were sown each in a plastic tray (7.5 in × 12.5 in × 2 in) using a sterilized potting mixture (Sunshine professional growing mix: Sun Gro Horticulture Canada Ltd., Agawam, MA, USA). All the trays were placed in popup cages (24 × 24 × 36 in, Biogentex Laboratories, Inc., TX, USA) inside the greenhouse conditions at 27 °C and RH 70%. Trays were monitored daily for germination for 70 days starting from the day after sowing until no further germination was observed for 5 consecutive days. Germination was measured in two ways; number of seeds germinated over total seeds planted (germination rate), and number of seeds germinated per week (speed of germination). After seedlings produced 2–4 true leaves, they were transplanted to square pots (4 × 4 × 6 in) inside the popup cages.

#### Growth traits

The seedlings were monitored for height and leaf count every two weeks after transplanting. The height was measured using a ruler (cm) and the number of fully developed leaves were counted. In addition, total shoot length was also measured before harvesting for root traits.

#### Root traits

Morphological characteristics of roots were measured from a total of 30 plants composed of both treatments: 15 mowed and 15 unmowed. These plants were randomly chosen from the transplanted seedlings, all at the same age post-transplanting (4 weeks after transplanting). The plants were cut at soil level to separate the shoots from the roots and then gently removed from their respective pots and placed on a 3-part strainer, where soil was gently washed off the roots. Image acquisition of roots was completed by placing washed roots submerged in water on a transparent tray and scanned with an EPSON Flatbed Scanner (EPSON Expression 11000XL 1.8 V3.49 3.49), part of the WinRHIZO package (Regent Instruments Inc., Quebec, Canada). WinRHIZO has been used to efficiently and precisely determine complex root parameters that are normally prone to human error^58^ WinRHIZO was used to digitize and quantify various root traits such as total root length (cm), area (cm^2^), the number of tips, forks, crossing (fine roots) and root volume (cm^3^), among others. Specific root length (m/g) was determined by dividing the total root length by total root dry biomass.

Detailed explanation of the variables measured are in Table 1.

#### Dry biomass

After root trait quantification, roots and shoots were dried at room temperature in brown paper bags for two days. After drying, the bags were placed in a drying oven (Quincy lab. INC, Fisher Scientific, USA) at 75 °C for 48 h, and dry mass was measured using an analytical balance (Accuris Dx W3101A-220, Mid Sci, Valley Park, MO, USA).

### Herbivory

#### Field herbivory

To determine whether mowed and unmowed plants experience similar herbivory levels in field, we did a field survey on ~ 10 plants per subpopulation and estimated herbivory levels on a 0–4 scale; 0 = 0%, 1 = 25%, 2 = 50%, 3 = 75% and 4 = 100% of the leaves damaged^59^. Since mowing continuously reduced leaf area, we restricted our estimation to the youngest 5–6 fully developed leaves to be consistent across treatments. In addition, we also estimated herbivory presence on a yes or no (0 or 1) binary scale as an additional line of data for herbivory in field. The same methodology was repeated for seedlings from the next generation, except the transplanted seedlings in pots were transported to field and placed in SNL populations as a pair (one mowed and one unmowed; 15 pairs) 1 m apart for 7 days, followed by damage and herbivore assessment as before.

#### Herbivory in lab

To determine whether mowing impacted plant response against specific herbivores, we followed up the field experiment with lab assays with three different herbivores: Tobacco hornworm (*Manduca sexta*), a generalist on Solanaceae, cow pea aphid (*Aphis craccivora*) a generalist aphid, and Texas Potato Beetle (*Leptinotarsa texana*), a specialist on SLN. These three herbivores are commonly found in the native SNL populations, and have been documented to successfully complete their life cycle on SNL.

##### *Manduca sexta* larval mass gain

*M. sexta* caterpillars were collected from the lab colony reared on a wheat germ based artificial diet (Frontier Scientific Services, Newark, DE, USA^60^). Two days old *M. sexta* eggs were placed on a 1 cm^3^ cubes diet inside a petri dish until they hatched. After hatching, first instar caterpillars were pre-weighed and placed on fully developed leaves of 4 weeks post transplanted seedlings of SLN. In this experiment, sixty SLN plants (thirty mowed and thirty unmowed) were used. The plants were not randomly selected, but chosen based on similarity in size, height, and number of leaves to reduce any confounding traits on herbivore mass gain. A coffee filter paper was wrapped around the potted plants around the midpoint of the stem, such that each plant was divided into two halves, with each half receiving one caterpillar each. Two first instar *M. sexta* caterpillars were placed on each plant, one caterpillar above the coffee filter and one caterpillar below the filter on a fully developed leaf and could feed continuously for 4 days. The caterpillars were starved for 4–6 h before the experiment to clear their gut. After 4 days, the caterpillars were removed, and post mass data was collected. Using the following equation mass gain was calculated: mass gain = (final mass − initial mass)/initial mass^61,62^.

##### *Aphis craccivora* population growth

*Aphis craccivora* used for the experiment was from a lab colony reared on multiple Solanaceae species. In this experiment a total of twenty-four plants; twelve mowed and twelve unmowed were used. For the population assay, three third instar aphid nymphs were transferred from the host plants to a young leaf of treatment plant using a paint brush and were allowed to grow and reproduce. The plants were separated and caged individually to minimize any accidental spread. Aphids were monitored and counted every five days and were counted twice. For both counts adults and nymphs were counted separately.

##### *Leptinotarsa texana* larval mass gain

Like *A. craccivora*, we used a lab colony of *L. texana* reared on Solanaceae species from individuals collected from SNL from the native populations in the Summer of 2019. We used newly molted second instar grubs for the experiment, pre weighed and placed on fully developed leaves of 15 mowed and 15 unmowed treatment plants. After four days, beetles were removed from the plant using a small paint brush and weighed on a balance (Accuris Dx W3101A-220, Mid Sci, Valley Park, MO, USA) for the second mass. Like *M. sexta*, mass gain was then calculated.

#### Trichome density

To examine how trichome density in offspring was influenced by mowing treatment imposed on maternal plants, we chose one leaf each from 10 randomly chosen offspring seedlings from mowed and unmowed parents. These leaves were cut near node of the plant to avoid any damage to the leaf, then using a hole punch two small disks (6 to 8 mm diameter) were cut for each leaf. Leaf disks were taped with carbon tape and then was placed on a 15 mm aluminum stage. To examine the trichomes in detail, we used a desktop scanning electron microscope (SNE-4500 M Plus Tabletop SEM; Nanoimages LLC, Pleasanton, California, USA). Images of abaxial and adaxial sides of leaves were taken at 60 × magnification with 5KV using SE detector. For each sample, we did the following measurements: trichomes on abaxial and adaxial surface, number of glandular vs non-glandular trichomes, and then for each sample, 10 random non glandular stellate trichomes were chosen and the number of individual spikes on them were counted. For counting, the scanning electron micrograph was saved as a .jpg file and each trichome was identified, labeled and counted^63^.

### Statistical analysis

For total fruits, seed mass, seeds/fruit and total seeds data, we used a General Linear Model (GLM) with Poisson distribution. Tukey comparisons were carried out to determine pairwise differences among the factors including sub-populations and mowing or unmowed treatments. Fruit diameter was analyzed using the non-parametric Kruskal–Wallis test to determine if mowed populations varied from unmowed, since data failed to meet normality assumptions even after transformation attempts. Plant height from mowed and unmowed sub-populations were collected twice and analyzed separately using Kruskal–Wallis tests. Damage assessment on 0–4 scale data was analyzed using Poisson regression. Seed germination data was also analyzed with a General linear Model with mowing (or unmowed control), GA (no GA control) treatment and week (week 0–9) as factors, followed by Tukey posthoc tests to tease apart pairwise comparisons. For analyses of root traits, we used a combination of two-tailed T tests and non-parametric Kruskal–Wallis tests based on the distribution of the data. Variables for which the data didn’t meet normality assumptions even after transformation were analyzed by Kruskal–Wallis tests. Specific root length was analyzed using two tailed t-tests. Transformed data was back transformed for reporting as means and for plots. In both analyses mowed/unmowed treatment was used as the predictor. For experimental design and analyses, each genet (individual plant) is considered as the unit of replication, for both parental generation (sub-populations) and seedlings. The detailed statistics are displayed in the table, and a few of the most relevant root traits of interest^64^ are displayed as plots (see Table 1). For field herbivore presence (yes or no) and herbivory scale (0–4) data analyses, we used binary logistic regression and ordinal logistic regression respectively with treatment (mowed /unmowed) as the predictor. P values were reported based on Wald’s test. To confirm that any preexisting variation in plant traits did not factor into field herbivory assessment, we also ran a t-test on plant height (Supplementary Data). *M. sexta* mass gain and *L. texana* mass gain was analyzed using the Kruskal–Wallis test due to non-normal distribution. *A. craccivora* population growth was analyzed by examining total aphids found (adults and nymphs) using a Poisson distribution fit model regression due to non-normal count data. Both treatment and replicate were used as predictors and P values were reported from Wald test*.* For trichomes we ran multiple analyses; total trichomes were analyzed using a 2-sample t-test, and a Two-way Anova was used for estimating whether the trichomes varied due to mowing treatment of leaf surface. Treatment (mowed/unmowed) and side (abaxial/adaxial) were used as factors. Similar to total trichomes, stellate and non-glandular trichome number, and number of spikes on stellate trichomes were also analyzed using 2 sample t-tests. All analyses were carried out using Minitab (Minitab Inc, State College, PA, USA) and plots were made using GraphPad Prism (LA Jolla, California, USA) software.

## Results

### Fitness traits (parents)

**Figure 1:**
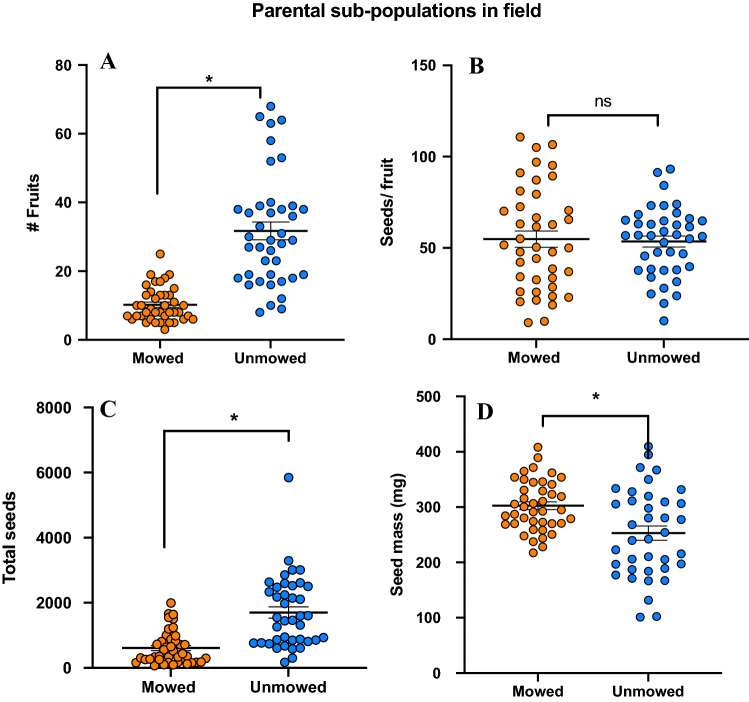
Results of field fitness traits (**A**) fruit set on mowed and unmowed locations (P < 0.001), (**B**) number of seed per fruit (Y-axis) (P = 0.809), (**C**) 100 seeds mass (Y-axis) (treatment P < 0.001), (**D**) total number of seeds (Y-axis) (P < 0.001). Means are shown by scale bars and asterisk denotes significantly different results at p < 0.05, while ns denotes non-significant results.

Analyses of total fruits production showed that unmowed genets produced significantly more fruits (GLM; F = 48.72; P < 0.001; Fig. 1A), and total seeds (Mean seeds × total fruits; GLM; F = 41.90; P < 0.001; Fig. 1B). However, we also found that there was no difference for fruit diameter (Kruskal–Wallis Test; P = 0.269; see Supplementary Fig. 1), and for mean number of seeds per fruits (GLM; F = 0.06; P = 0.809; Fig. 1C). Surprisingly, when we measured 100 seed mass, we found that seeds from mowed genets were significantly heavier than unmowed genets, suggesting that these embryos may be better fit (GLM; F = 3.35; P < 0.001; Fig. 1D), a question we addressed with the germination assays.

### Seed germination

**Figure 2:**
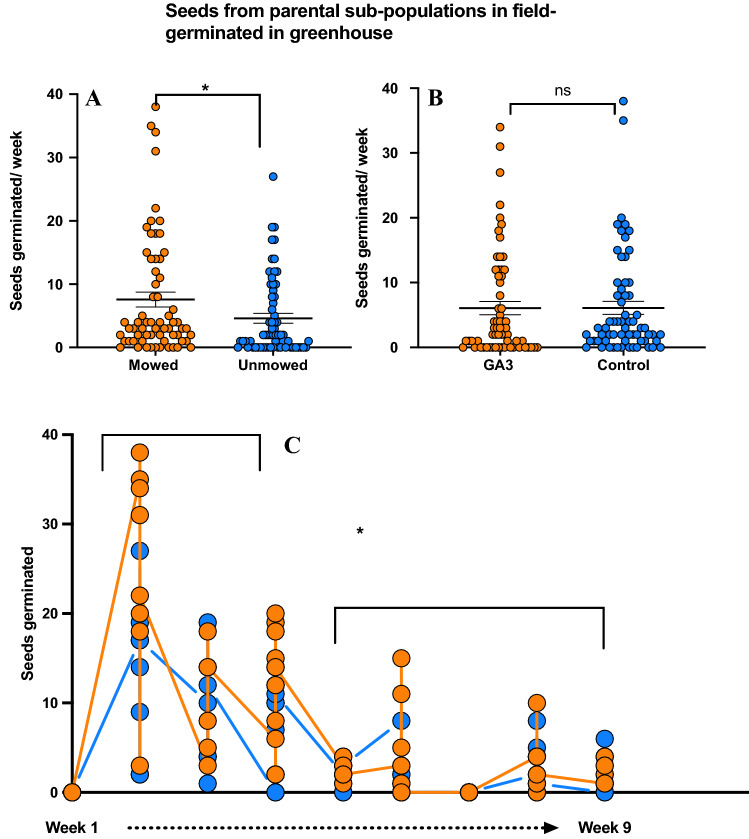
Results of seed germination in green house (**A**) seeds germinated per week on mowed and unmowed plants (P < 0.002), (**B**) seeds germinated per week with GA_3_ treatment and control (P = 0.974), (**C**) Seeds germinated over time (early vs late season) (treatment P < 0.006; GA_3_ P = 0.977), mowed and GA_3_ (orange) and unmowed and control (blue) plants. Means are shown by scale bars and asterisk denotes significantly different results at P < 0.05, while ns denotes non-significant results.

Following our fitness traits experiments, seed germination showed that seeds from mowed maternal plants had significantly more germination than from unmowed (GLM; F = 9.85; P < 0.002: Fig. 2A). However, the phytohormone GA3 had no significant effect on germination rate for mowed and unmowed populations (GLM; F = 0.00; P = 0.974; Fig. 2B). Like other weed species, we also found significantly more seeds germinated during the early season than in the late season (GLM; F = 95.33; P < 0.001) with most of the germination taking place in the first five weeks (Fig. 2C).

### Growth (offspring)

**Figure 3:**
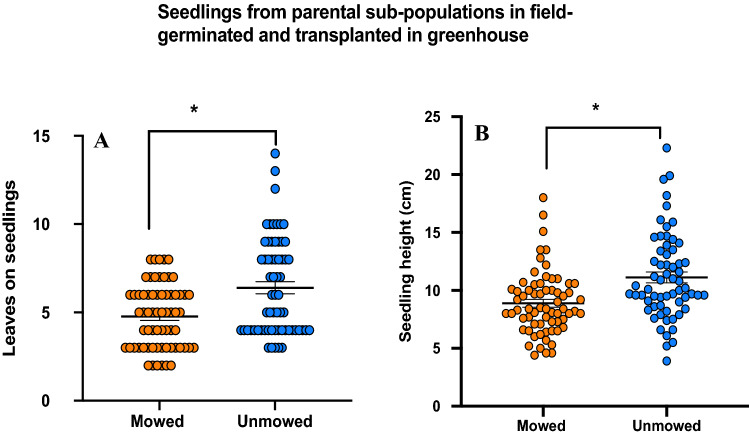
Results of seedlings health and vigor (**A**) number of leaves on seedlings on mowed and unmowed plants (P < 0.001), (**B**) seedling height (cm) on both treatments (P < 0.001) mowed (orange) and unmowed (blue) plants. Means are shown by scale bars and asterisk denotes significantly different results at P < 0.05, while ns denotes non-significant results.

Transplanted seedlings from the germination experiment were also monitored for health and vigor. We found that seedlings from unmowed parents had significantly more leaves per seedling (Poisson distribution fit model regression; Chi-square = 15.16; P < 0.001) and were also significantly taller than mowed plants (Kruskal–Wallis test; t = − 3.81; P < 0.001), suggesting better growth and developmental traits in unmowed seedlings (Fig. 3A,B).

### Field damage (parental generation)

**Figure 4:**
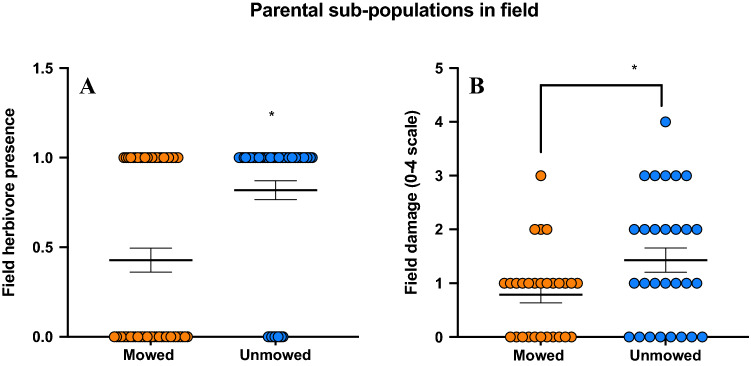
Results of seedling herbivory in field: (**A**) herbivore presences on mowed and unmowed plants (P < 0.001; binary logistic regression; Wald’s test), (**B**) damage by insects on both treatments (scale 0–4) (P = 0.037; ordinal logistic regression; Wald’s test) mowed (orange bar) and unmowed (blue bar) plants. Means are shown by scale bars and asterisk denotes significantly different results at p < 0.05.

Analysis of herbivory data from field showed that significantly more herbivores were present on unmowed plants in comparison to mowed plants (Binary logistic regression; Chi-square = 16.92; P > 0.001; Fig. 4A). Consequently, damage done by herbivores was also significantly more on unmowed plants (Ordinal logistic regression; P-value = 0.037; Fig. 4B).

### Herbivory in lab (offspring)

**Figure 5:**
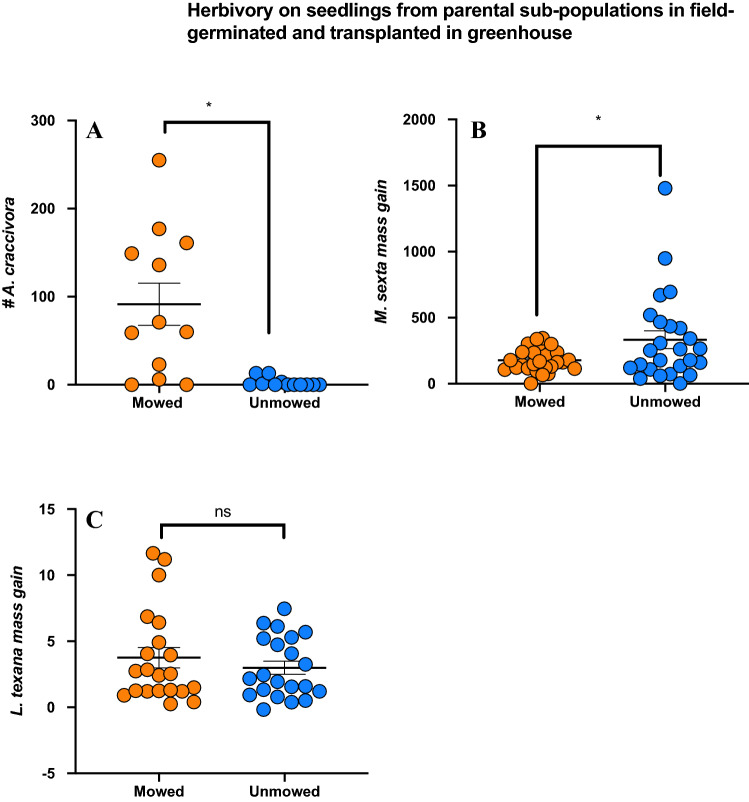
Results of herbivory in lab results from 3 herbivores (**A**) number of *A. craccivora* (Y-axis) (P < 0.001; Poisson distribution fit model regression), (**B**) mass gain of *M. sexta* caterpillars (Y-axis) (P = 0.022; Kruskal–Wallis test) and (**C**) mass gain of *L. texana* (Y-axis) on mowed (orange bar) and unmowed (blue bar) plants (P = 0.715; Kruskal–Wallis test). Means are shown by scale bars and asterisk denotes significantly different results at p < 0.05, while ns denotes non-significant results.

Field damage results were further confirmed by herbivory experiments conducted in lab. We found the number of *A. craccivora* on mowed plants to be significantly higher than on unmowed plants (Poisson distribution fit model regression; P-value < 0.001; Fig. 5A). However, our mass gain experiments of *M. sexta* show significantly lower mass gain on mowed plants than on unmowed plants (Kruskal–Wallis test; H = 5.22; P-value = 0.022) (Fig. 5B). On the other hand, we found no significant difference in mass gain of *L. texana* on both mowed and unmowed plants (Kruskal–Wallis test; H = 0.13; P-value = 0.715) (Fig. 5C). Therefore, we speculate higher induction of jasmonic acid (JA) signaling pathway in mowed treatments, which is induced in plants upon attack by chewing insect pests. These results are akin to JA-SA (Salicylic acid pathway induced in plants upon attack by sucking insect pests) pathway negative crosstalk, where induction of one pathway downregulates the other.

### Trichomes (offspring)

**Figure 6:**
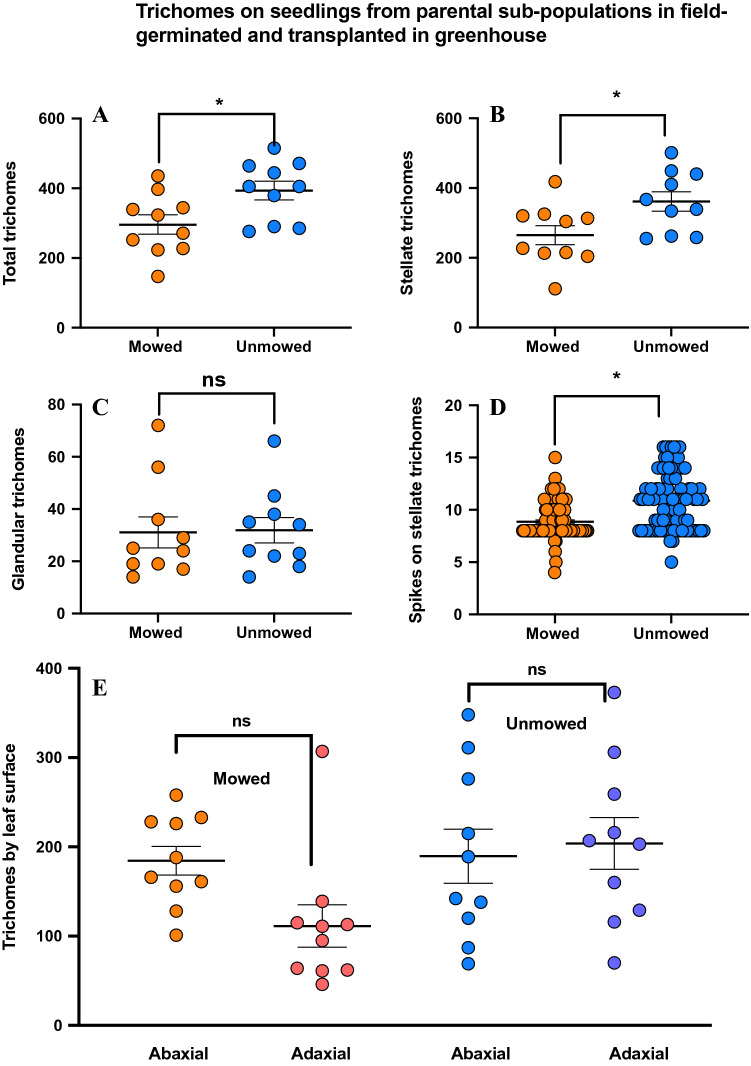
Results of leaf trichome density: (**A**) number of trichomes on leaves for mowed and unmowed treatments (Y-axis) ((Two sample T-test; t = − 2.53; P-value = 0.02), (**B**) compares mean number trichomes on abaxial and adaxial side of the leaf for both mowed (Y-axis) (Two-way Anova; F = 1.27; P-value = 0.26) and unmowed (Y-axis) (Two-way Anova; F = 3.50; P-value = 0.06) treatments respectively, (**C**) mean number of stellate trichomes on leaves for mowed and unmowed treatments (Y-axis) (Two sample T-test; t = − 2.47; P-value > 0.02), (**D**) mean number of glandular trichomes on leaves for mowed and unmowed treatments (Y-axis) (Two sample T-test; t =  − 0.10; P-value = 0.918), (**E**) number of spikes on stellate trichomes for mowed and unmowed treatments (Y-axis) (Two sample T-test; t = − 6.26; P-value < 0.00). Means are shown by scale bars and asterisk denotes significantly different results at P  < 0.05, while ns denotes non-significant results.

Contrary to our expectations, our results show that unmowed plants have significantly more trichomes than mowed treatments (Two sample T-test; t = − 2.53; P-value = 0.02) (Fig. 6A). We followed this by examining the abaxial and adaxial side of the leaves for both treatments. However, there was no significant differences in mean number of trichomes per side (Two-way Anova; F = 1.27; P-value = 0.26) (Fig. 6B) in either of the treatments (Two-way Anova; F = 3.50; P-value = 0.06) (Fig. 6B). SLN has both glandular and non-glandular (stellate) trichomes, dominated by non-glandular stellate trichomes. Similar to trichome density we also found that unmowed treatment had significantly more stellate trichomes (Two sample T-test; t = − 2.47; P-value > 0.02) (Fig. 6C), while no difference was found between treatments for glandular trichomes (Two sample T-test; t = − 0.10; P-value = 0.918) (Fig. 6D). Using enhanced measurement features of the tabletop SEM, we also examined detailed morphology of non-glandular (stellate) trichomes and found that the seedlings from unmowed treatment also had significantly more spikes on their trichomes (Two sample T-test; t = − 6.26; P-value < 0.00) (Fig. 6E).

### Root traits

**Figure 7:**
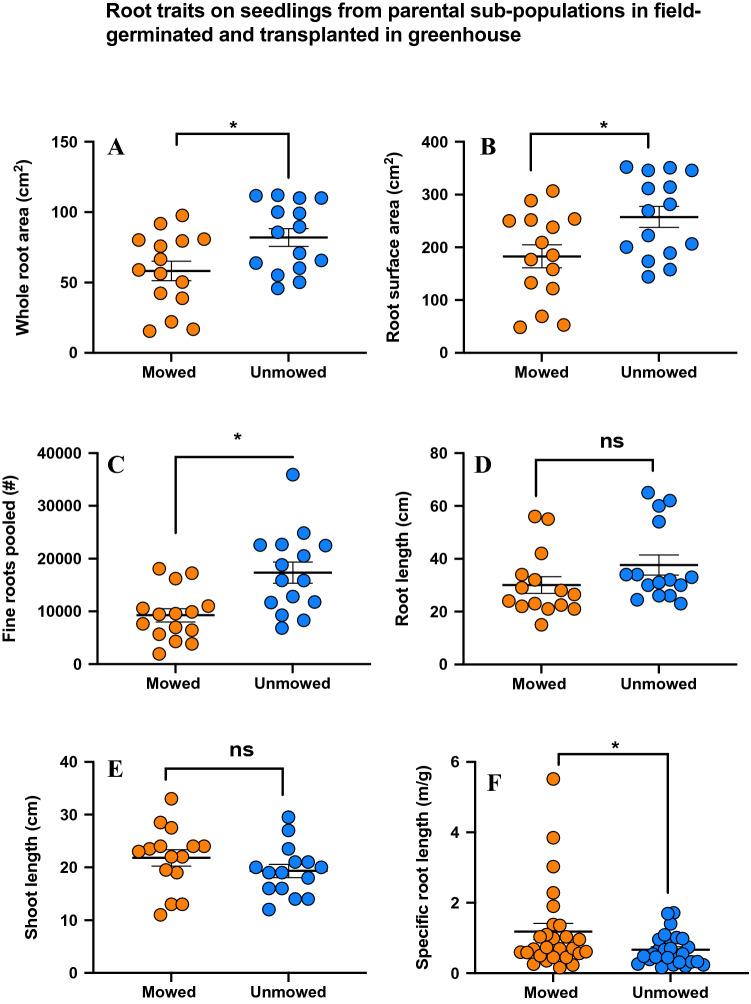
Results of six major root traits show: (**A**) Whole root area (P < 0.108; Kruskal–Wallis tests), (**B**) Root surface area (P = 0.017; Kruskal–Wallis test), (**C**) Fine roots pooled (P < 0.0002; Kruskal–Wallis tests), (**D**) Root length (P = 0.004; Kruskal–Wallis tests), (7E) Shoot length (P = 0.225; Kruskal–Wallis tests), (7F) Specific root length (P < 0.049; two tailed T test) on mowed (orange bar) and unmowed (blue bar) plants. Means are shown by scale bars and asterisk denotes significantly different results at p < 0.0, while ns denotes non-significant results 5.

In addition to above ground traits, we also examined below-ground root traits and their differences between seedlings from mowed and unmowed parents. There were no significant differences between the two groups in root (Kruskal–Wallis tests; t = − 0.130; P < 0.108) (Fig. 7A, Table 1) and shoot (Kruskal–Wallis tests; t = 1.24; P = 0.225) (Fig. 7B, Table 1) length. However, we found that the major root traits such as whole root area (Kruskal–Wallis tests; t = − 2.54 P = 0.017) (Fig. 7C, Table 1), root surface area (Kruskal–Wallis tests; P = 0.017) (Fig. 7D, Table 1), and fine roots (Kruskal–Wallis tests; t = − 3.42 P < 0.0002) (Fig. 7E, Table 1), were significantly higher in roots from seedlings of unmowed parents than their mowed counterparts. Interestingly, specific root length (SRL), a key root trait in resource acquisition, was significantly higher on mowed when compared to the unmowed seedlings (Two tailed T test; t = 2.02; P < 0.049) (Fig. 7F, Table 1).

## Discussion

In this study we examined how disturbance (mowing) affects reproductive fitness traits, defenses, and their cascading effects on SLN over two growing seasons. Collectively, we found that although mowing reduces reproductive fitness by removing photosynthetic area, mowing also leads to local adaptation for fitness and defense traits in both parental and offspring generations, potentially leading to become a superweed. More importantly, we also found that these differences are not similar for all the traits we measured. Enhanced fitness and defense traits in the offspring, from mowed parents indirectly suggest that local disturbance can lead to better fit seedlings with a possibility of making species like SLN a superweed, adding another layer of complexity in understanding its invasion and management^50^.

While examining fitness traits, we found that unmowed genets were taller and produced significantly more fruits and total seeds (total fruits X seeds/fruit) than their mowed counterparts (Fig. 2), confirming that these genets can flourish in anthropogenically undisturbed environments^65^. In contrast, while unmowed genets produced more fruits, the seed mass of mowed individuals was significantly higher (Fig. 2C). It has been well understood that heavier seeds tend to germinate more and rapidly, therefore seed mass is considered a strong indicator of fitness^66^. This is particularly important for a weed species such as SLN that colonize agricultural land, pastures and areas that are prone to constant disturbance. Our data clearly demonstrates that higher seed mass can have a benefit by improving progeny fitness, as documented elsewhere^67^ again, alluding to local adaptation.

As a consequence of heavier seeds on mowed plants, we found that germination was more frequent and more rapid when compared to unmowed plant seed progeny. We also introduced the phytohormone GA_3_ to test if the germination accelerant phytohormone treatment can differentially influence germination rate^68^, but surprisingly, found that GA_3_ has no effect on germination rates on mowed or unmowed treatments. We speculated that if GA_3_ brought the germination rates of unmowed plants up to par with the mowed ones, it would suggest that enhanced GA_3_ signaling in the mowed plants can possibly be induced by mowing. Clearly, enhanced germination is the result of the environmental stress through mowing, possibly independent of any genetic effects, an area that needs to be examined further. We also estimated the germination cycle of SLN in its native range. We show that SLN seeds have around 50% germination rate and tend to germinate the most within the first 5 weeks of seeding, as opposed to later in the season (Fig. 3). This coincides with a study on germination timings of another (of many) weed species, where *Ambrosia artemisiifolia* L., *Ambrosia trifida* (rag weed), and *Polygonum pensylvanicum* L., (Knotweed) all had large flushes of germination in the first 5 weeks from their planting, followed by very little to no germination after^69^. Gioria and Pysek, 2016 also found a strong tendency for invasive plants to germinate earlier and faster than their native counterparts. In addition, since germination rate also reflects local adaptation^70^ to changes in the environment^71^, our data complements the list of studies on local adaptation, with traits measured in two generations of growth. For a weed species that tends to undergo constant disturbance and possible extinction of founding populations, a rapid and relatively high germination rate make it a grave concern for conservation, invasion and management policies^72,73^.

Interestingly though, enhanced germination rates and faster germination didn’t translate into better growth traits in mowed offspring. We found that seedlings from unmowed parents were taller and had more leaves, suggesting that mowing-induced trait enhancement is possibly limited to germination, rather than cascading throughout the growing season^74, 75^. Additionally, weeds like SNL have higher root investment, since rhizomes are a major reproductive strategy, ensuring fitness. Similar to growth and fitness traits, we found that unmowed plants had higher values of various metrics of root biomass, including root area and number of fine roots, but a key metric of the cost–benefit ratio of roots, specific root length, was significantly higher in offspring of mowed plants (Table 1). Specific root length has been associated with an enhanced ability to acquire nutrients and is known to be independent of other plant trait economics spectra^76,77^. This is a significant result as it clearly shows that while total available resources are limited, mowed offspring construct fine roots more efficiently, and thus may be able to partially offset these negative effects, giving them enhanced ability to acquire water and nutrients at a given size. Additionally, this ability can be critical in limited resource environments. Their potential implications for invasion success, and consequential effects on plant diversity under different land management regimes^78^ can shed light into how invasive weed species can be successful under resource limitation^79^.

A large body of previous research has investigated the defense mechanisms in plants against insect herbivory in weeds^56,80–82^, and how these interactions are modulated by resource availability, evolutionary history, and breeding status^21,83–85^. In both years, a significant amount of our experimentation was carried out on herbivory and plant defenses. We hypothesized that mowing, a mode of mechanical wounding will enhance defenses and thereby negatively impact herbivores^63^ that feed on SLN. In the parental generation, we found that mowed plants suffered lower damage in the field. The major herbivore of SLN in our subpopulations was Texas potato beetle (*Leptinotarsa texana*) grubs and adults, other herbivores present were tobacco hornworm (*Manduca sexta*), cowpea aphid (*Aphis craccivora*), and eggplant tortoise beetle (*Gratiana pallidula*). Even more interesting was that the reduced herbivory was consistent in offspring when they were exposed to herbivores in an area close to our subpopulations. Clearly, mowing (damage) in parental generation enhanced offspring defenses and they possibly had higher constitutive defenses that reduced both herbivore incidence and herbivory levels^44^. For example., it has been shown that higher alkaloid production in tall fescue (*Festuca arundinacea*) and perennial ryegrass (*Lolium sp.*) post mowing resulted in lower herbivore damage, supporting the idea these disturbance help plants to withstand and maybe even better defend against herbivores^86,87^.

We found that our lab experiments on herbivory results were species specific; *M. sexta* (chewing herbivore; feeds on most Solanaceae members) gained less mass on mowed plants inflicting less damage to these plants than unmowed plants (Fig. 5B). However, *L. texana* (chewing herbivore, co-evolved and feeds exclusively on SLN^88^ mass gain was similar in both mowed and unmowed plants (Fig. 5C). On the other hand, *A. craccivora* (sucking herbivore, generalist aphid) population fared significantly better on mowed plants (Fig. 5A). In general, aphids induce SA pathway (salicylic acid phytohormonal signaling that provides resistance to plants against sucking insect pests and pathogens; 90) while chewing herbivores induce JA (Jasmonic acid) pathway in plants. Results from herbivory experiments are consistent with the JA and SA pathway negative crosstalk. Plenty of studies have reported negative crosstalk of JA suppressing SA action^89,90^. Traw et al. also found suppression of SA due to JA in Wassilewskija wild type of *Arabidopsis thaliana *which increased their susceptibility to *Pseudomonas syringae *^89^. We speculate that increased constitutive defenses under the JA pathway enhanced defenses against herbivores in general (as observed in field), and more specifically against *M. sexta* in lab and field damage on the seedlings. Consequently, JA mediated SA suppression possibly led to mowed SLN being susceptible to sucking herbivore A. craccivora. However, the most important herbivore that damages SLN- *L. texana*, a potential biocontrol agent^91^ was unaffected by mowing, clearly suggesting that regardless of any enhanced defenses due to mowing, the co-evolved specialist herbivore was able to continuously feed and develop, as documented in other systems^92,93^. For example, Yang et al., looked at *Triadica sebifera* in its native (Asia) and invasive habitat (USA). Using two generalist and one specialist herbivores, they found that even though the chemical composition of flavonoids and tannins changed in their respective habitat, the specialist fed more and consequently had better growth when compared to the generalist herbivores^94^. Similarly, Blair and Wolfe, have shown that plants introduced to new environmental conditions found to have faster germination, growth and enhanced reproduction but invest less in defense traits due to reduced herbivory pressure and enemy free space^95^. Our results are opposite (lower height and reduced number of leaves in mowed plants) is possibly due to the need of increased investment in defense traits due to higher herbivory and continuous mowing.

Finally, the variation in herbivore response to mowing in offspring in laboratory conditions and field conditions lead us to ask whether plant defenses correlate with these herbivore growth traits. Our comprehensive examination of the trichome morphology of SLN allowed us to address this directly. Using a series of manipulative experiments, we have previously documented pre and post ingestive roles of trichomes as a plant defense in Solanum spp- *M. sexta* system^60, 96,97^. Surprisingly, our results showed that offspring from mowed parents had lower trichome density (stellate, the major trichome type), and that they also had lower number of individual spikes on them. Trichomes have been well documented to be an effective defense against herbivores, by either restricting their access^97,98^, movement^99,100^ and in many cases being toxic to them^60,83,96^. We speculate that although trichomes are thought to be primarily regulated by JA pathway, other phytohormones including GA_3_, Cytokinins, SA and Ethylene also plays key roles in both initiation and branching^101^. Our data clearly shows that the interplay of JA, SA mediated defenses, herbivore feeding and trichomes are far more complicated. It would be interesting to identify and quantify secondary metabolites (alkaloids, and plant volatiles), signaling compounds (phytohormones) and their gene expression to tease apart these effects, and to examine potential trade-offs between chemical and structural defenses and herbivory in this species.

Taken together, our data from both parental and offspring generations affected by mowing pressure strongly supports the idea that environmental anthropogenic disturbances significantly influence growth and fitness traits and leads to cascading effects from parent to offspring that could lead to possible super weeds, independent of herbicide resistance. Moving forward, the role of epigenetics^102–104^ in offspring trait expression should be explored further and will be the subject of future work in SLN.

## Supplementary Information

Supplementary Information
