# Supplementary material for: Local adaptation to continuous mowing makes the noxious weed Solanum elaeagnifolium a superweed candidate by improving fitness and defense traits
Source: Sci Rep. 2021 Mar 23;11:6634. doi: 10.1038/s41598-021-85789-z (PMC7988165; doi:10.1038/s41598-021-85789-z)
Supplement: Supplementary file 1 — Supplementary Information [file 41598_2021_85789_MOESM1_ESM.pdf]

Local adaptation to continuous mowing makes the noxious weed *Solanum elaeagnifolium* a superweed candidate by improving fitness and defense traits.

Jesus Chavana[1], Sukhman Singh[1] Alejandro Vazquez [1], Bradley Christoffersen[1], Alexis Racelis[1, 2], and Rupesh R. Kariyat[1, 2]

[1] Department of Biology, The University of Texas Rio Grande Valley, Edinburg, Texas 78539.

[2] School of Earth, Environmental and Marine Sciences, Edinburg, Texas 78539

Author for correspondence: Rupesh.kariyat@utrgv.edu

Supplementary Figures and Tables

Table 1. Location coordinates of mowed and unmowed sub-populations of SLN used for this study

Location	*Latitude*	*Longitude*	*Treatment*
Edinburg	26.313476	-98.145862	Mowed
Edinburg	26.306722	-98.145631	Mowed
McAllen	26.27475	-98.223654	Unmowed
McAllen	26.168613	-98.313712	Unmowed

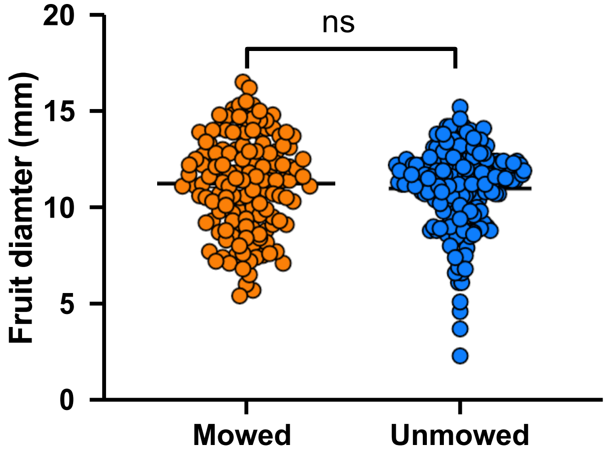

Supplementary figure 1: Results of fruit diameter from plants collected from 4 mowed and umowed sub-populations(P=0.269).
